# Case Report: Clinical analysis and literature review of two cases of high-grade adult-type cervical embryonal rhabdomyosarcoma

**DOI:** 10.3389/fonc.2026.1856828

**Published:** 2026-08-11

**Authors:** Wenyu Song, Tian Li, Yulong Chen, Qin Yao, Kejuan Song

**Affiliations:** 1 Department of Obstetrics and Gynecology, The Affiliated Hospital of Qingdao University, Qingdao, China; 2 Department of Obstetrics and Gynecology, Qingdao University Medical College, Qingdao, China

**Keywords:** adult-onset tumor, cervical neoplasms, distant metastasis, embryonal rhabdomyosarcoma, immunohistochemistry, multidisciplinary treatment, prognosis

## Abstract

**Background:**

Embryonal rhabdomyosarcoma (ERMS) predominantly occurs in children; primary cervical ERMS in adults is exceedingly rare and readily misdiagnosed, as its typical presentations—abnormal vaginal bleeding and a cervical mass—closely resemble gestational trophoblastic disease and uterine sarcomas.

**Case report:**

We present two cases of high-grade cervical ERMS in reproductive-age women; both presented \with abnormal vaginal bleeding and were diagnosed with stage IVB disease with regional lymph node metastasis at initial admission. Case 1 was complicated by synchronous bilateral pulmonary and multiple osseous metastases, while Case 2 exhibited a massive cervical tumor with extensive lymphatic dissemination. The two patients received distinct multimodal treatment regimens: one underwent primary surgery followed by adjuvant chemotherapy; the other received interventional embolization, neoadjuvant chemotherapy, radical resection, sequential chemoradiotherapy, and exploratory targeted maintenance therapy.

**Conclusion:**

Adult cervical ERMS is highly aggressive and prone to early distant spread. Immunohistochemical markers are mandatory to confirm the rhabdomyogenic origin of this tumor. For patients with advanced disease, multimodal regimens combining neoadjuvant chemotherapy, radical resection (when surgically feasible), and adjuvant chemoradiotherapy may serve as a primary comprehensive approach to potentially prolong overall survival. In patients with actionable somatic alterations identified through comprehensive molecular profiling and formal HRD testing, individualized exploratory targeted therapy may be explored only in highly selected patients. Further clinical investigations are warranted to refine treatment strategies and improve long-term outcomes.

## 1 Introduction

Embryonal rhabdomyosarcoma (ERMS) is an aggressive soft tissue sarcoma of striated muscle precursor or primitive mesenchymal cell origin. It predominantly affects children and adolescents; adult cases are rare, and primary cervical ERMS is exceptionally uncommon (1, 2). Typical presentations include abnormal vaginal bleeding, uterine enlargement, and a cervical mass (3, 4). The tumor is highly invasive with marked metastatic potential; most patients present at an advanced stage with lymphatic and hematogenous spread, portending a far poorer prognosis than in pediatric disease. Standardized consensus recommendations exclusively for adult cervical ERMS have not yet been formulated, and clinicians refer to pediatric RMS and general soft tissue sarcoma guidelines for treatment reference (5, 6).

This study retrospectively analyzed two patients with high-grade cervical ERMS admitted to our hospital, summarizing their clinical manifestations, pathological features, treatment courses and survival outcomes, combined with a literature review, to provide clinical references for the identification and standardized management of rare adult genital tract ERMS.

## 2 Case summary

### 2.1 Case 1

**Figure 1:**
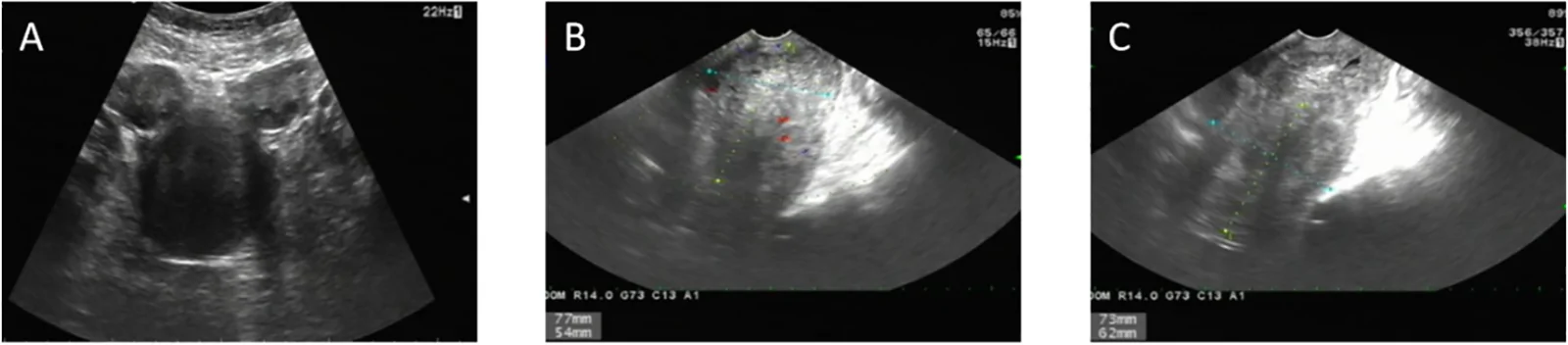
Preoperative gynecological ultrasound of Patient A. **(A–C)** Transvaginal ultrasound showing a 7.0 × 5.4 cm ill-defined heterogeneous cervical lesion with internal honeycomb cystic changes and hypervascularity on CDFI.

A 26-year-old woman presented to the Affiliated Hospital of Qingdao University on May 19, 2017, with a one-month history of vaginal bleeding. Diagnostic curettage had been performed at an outside institution one week prior. Gynecological examination revealed a grape-like mass, approximately 3 cm in diameter, protruding from the cervical os; the uterus was anteverted, enlarged to the size of a 2.5-month gestation, regular in contour, of moderate consistency, and freely mobile; no cervical motion or adnexal tenderness was elicited. Laboratory tests: β-hCG < 0.10 mIU/mL (normal < 3.0 mIU/mL); lactate dehydrogenase (LDH) 126 U/L (normal 120–250 U/L). Ultrasound demonstrated an irregular echogenic area measuring approximately 7.0 × 5.4 cm in the lower uterine segment and cervical canal, with ill-defined borders, internal honeycomb-like anechoic spaces, and abundant vascularity (**Figure 1**). Chest CT revealed multiple bilateral pulmonary metastases. Pathological review of the curettage specimen at our hospital showed vesicular, grape-like structures with areas of mucinous change and dense clusters of small cells exhibiting round, hyperchromatic nuclei. Immunohistochemistry: CKpan (−), β-HCG (−), LCA (−), GATA3 (−), MyoD1 (focal +), Myogenin (+), S100 (−), Syn (−), CD10 (−), Ki-67 ~90%. The findings were consistent with a malignant tumor, highly suspicious for embryonal rhabdomyosarcoma (botryoid subtype). PET/CT demonstrated a metabolically active cervical primary (SUVmax 3.7), hypermetabolic pelvic and retroperitoneal lymph nodes, and low-activity bilateral pulmonary nodules. Whole-body bone scintigraphy confirmed multifocal skeletal metastases involving the ribs and thoracolumbar spine.

**Figure 2:**
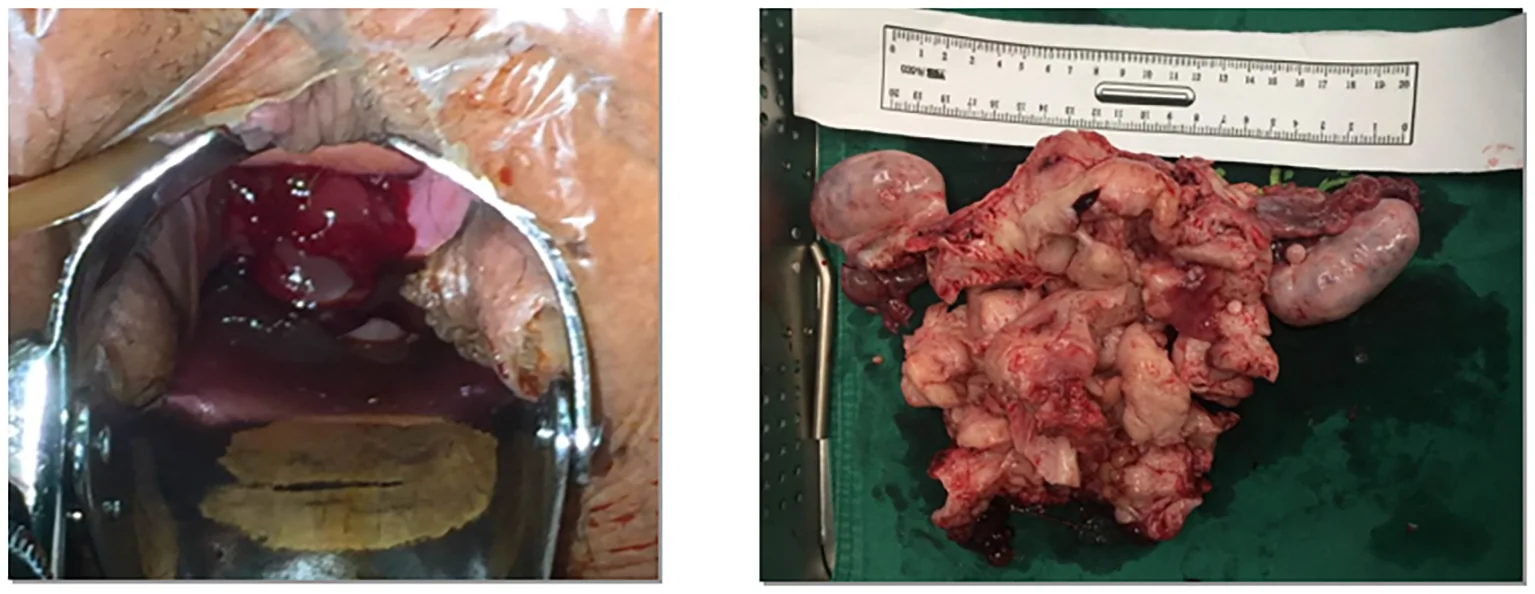
Findings of the gynecological examination and postoperative gross specimen of Patient A.

Following comprehensive assessment, surgical indications were established. On June 9, 2017, total hysterectomy, bilateral salpingo-oophorectomy, pelvic and para-aortic lymphadenectomy, and omentectomy were performed. Postoperative pathology confirmed a malignant small round cell tumor consistent with embryonal rhabdomyosarcoma. The tumor infiltrated nearly the full thickness of the cervical muscular wall with vascular tumor emboli (+); the vaginal resection margin was uninvolved; the endometrium, bilateral ovaries, and fallopian tubes were free of tumor (**Figure 2**). Immunohistochemistry: MyoD1 (weak +), Myogenin (+), Desmin (−), LCA (−), CKpan (−), Vimentin (+), Syn (−), CgA (−), CD99 (−), S100 (−), Ki-67 ~80%. Lymph node metastases were identified in the left para-aortic (1/1), left pelvic (3/8), right pelvic (5/10), and right para-aortic (4/9) regions; no tumor emboli were found in the ovarian vessels; the omentum was uninvolved. Ascitic fluid cytology was negative for malignancy. Postoperative diagnosis: Cervical embryonal rhabdomyosarcoma with extension to the lower uterine segment and endometrium, stage IVB (T3N2M1); secondary bilateral pulmonary and lymph node (pelvic, para-aortic) metastases; history of cesarean section.

The patient recovered uneventfully. Following multidisciplinary team evaluation, she received five cycles of vincristine, actinomycin D, and cyclophosphamide. Due to progression of pulmonary metastases, the regimen was switched to one cycle of paclitaxel plus carboplatin, followed by one cycle of docetaxel plus gemcitabine; neither regimen produced a response. In December 2017, multiple bone metastases developed. The patient subsequently experienced severe myelosuppression and poor tolerance to further chemotherapy. She was discharged at the family’s request, with follow-up continuing until February 2018.

### 2.2 Case 2

A 31-year-old woman presented to the Affiliated Hospital of Qingdao University in March 2024 with a two-month history of heavy menstrual bleeding. Her surgical history included tubal adhesiolysis and cesarean section. Gynecological examination revealed a cervix that was difficult to expose, firm, and confluent with the uterine body; the uterus was anteverted, enlarged to 3–4 months’ gestational size, firm, fixed, and non-tender. Laboratory tests: β-hCG < 0.10 mIU/mL; lactate dehydrogenase (LDH) 662 U/L. Pelvic ultrasound detected a large, ill-defined hypoechoic cervical mass measuring 11.6 × 9.4 × 9.4 cm with abundant vascular perfusion. Pelvic MRI confirmed a lesion with T1-isointense, T2-hyperintense, and diffusion-restricted signal, with a maximum diameter of 130 mm, highly suggestive of malignancy. PET-CT demonstrated hypermetabolic activity in the uterine body and cervical mass (SUVmax 16.9), as well as multiple enlarged, hypermetabolic lymph nodes (SUVmax 15.7) along the bilateral iliac vessels, retroperitoneal aorta, and inferior vena cava (below L3); a small hypermetabolic nodule was noted in the right paracolic gutter (SUVmax 2.1). These findings suggested a malignant uterine tumor with lymph node and peritoneal metastases. Diffuse, heterogeneous hypermetabolism was also observed in the medullary cavities of the axial skeleton, pelvis, and proximal extremities (SUVmax 10.7) without CT evidence of bone destruction, raising high suspicion for marrow involvement.

On March 12, 2024, ultrasound-guided transvaginal fine-needle aspiration biopsy of the pelvic mass was performed. Pathology revealed a small round cell malignant tumor; immunohistochemistry confirmed rhabdomyosarcoma (embryonal type): LCA (−), CKpan (−), CD56 (+), Syn (−), CgA (−), Ki-67 (~90%), INI-1 (+), SMARCA4 (BRG1) (+), Vimentin (+), Myogenin (+), MyoD1 (+), Desmin (+), CD43 (−), MPO (−).

**Figure 3:**
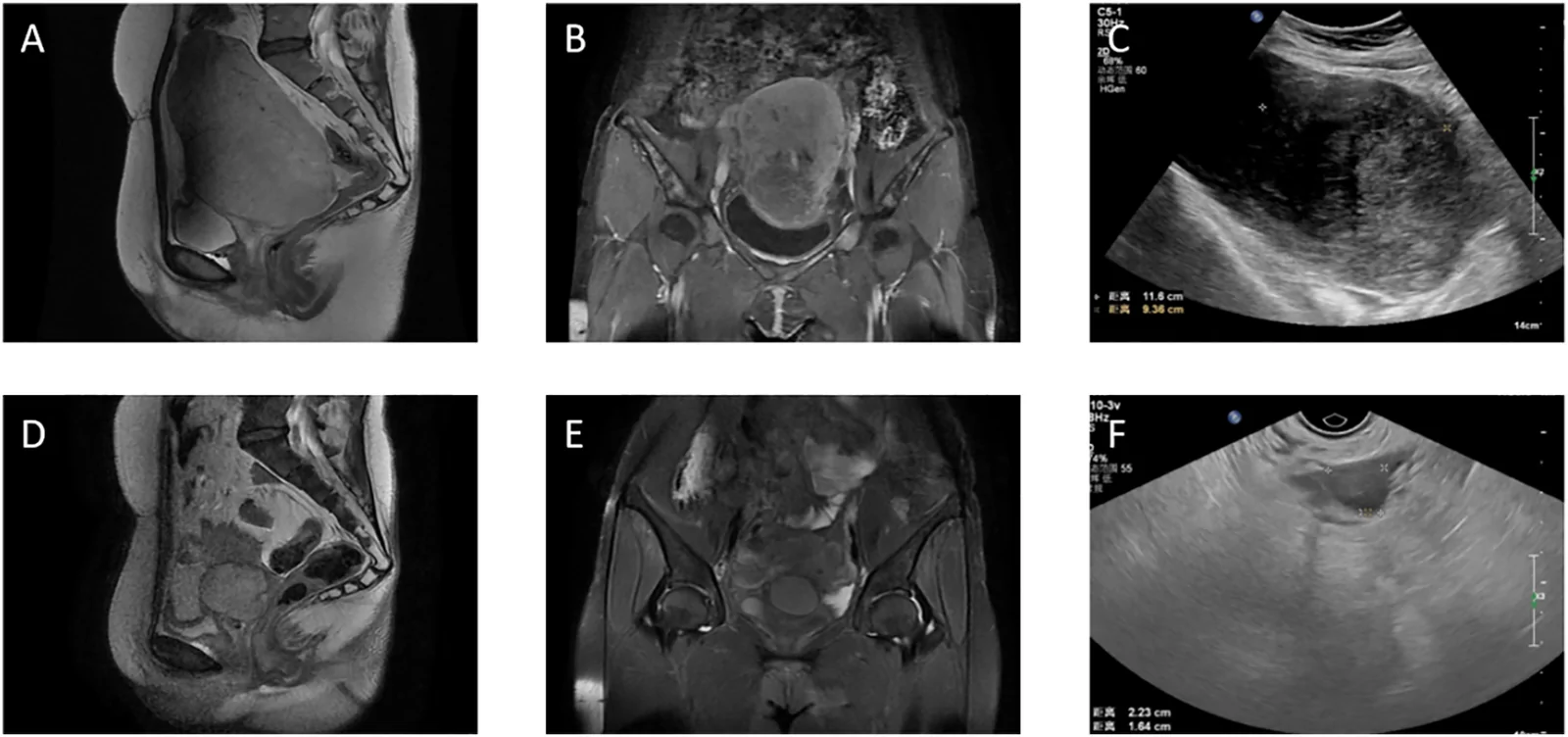
Comparison of pelvic MRI and gynecological ultrasound before and after neoadjuvant chemotherapy in Patient B. **(A, B)** Pre-neoadjuvant chemotherapy pelvic MRI showed a mass-like lesion in the uterine body and cervix with isointense T1 and hyperintense T2 signals; DWI demonstrated high signal intensity, with a maximum cross-sectional area of approximately 83 mm × 107 mm × 130 mm; **(C)** Pre-neoadjuvant chemotherapy gynecological ultrasound showed a 11.6 × 9.4 × 9.4 cm hypoechoic mass in the lower uterine segment and cervix, with indistinct borders and an irregular shape; CDFI: relatively rich intravascular blood flow signal; RI: 0.60; **(D, E)** Pelvic MRI after neoadjuvant chemotherapy showed a mass-like lesion at the cervix with T1-equivalent and T2-high signal intensity; DWI demonstrated slightly high signal intensity; maximum cross-sectional dimensions were approximately 48 mm × 42 mm × 44 mm; **(F)** Post-neoadjuvant chemotherapy gynecological ultrasound shows a hypoechoic mass in the lower uterine segment and anterior cervical lip, measuring 5.0 × 4.5 × 3.4 cm, with relatively well-defined borders and an irregular shape; CDFI: punctate and linear blood flow signals are observed peripherally.

Due to persistent vaginal bleeding, bilateral uterine artery embolization was performed on March 14, 2024.Following multidisciplinary team evaluation, the tumor was deemed unresectable in a single-stage procedure. The patient received four cycles of neoadjuvant chemotherapy with the VAC/VI regimen (discontinued due to severe delayed diarrhea after VI). Post-chemotherapy, LDH normalized to 207 U/L, and ultrasound demonstrated tumor shrinkage to 5.0 × 4.5 × 3.4 cm; whole-body bone scintigraphy showed no definite metastases (**Figure 3**).

**Figure 4:**
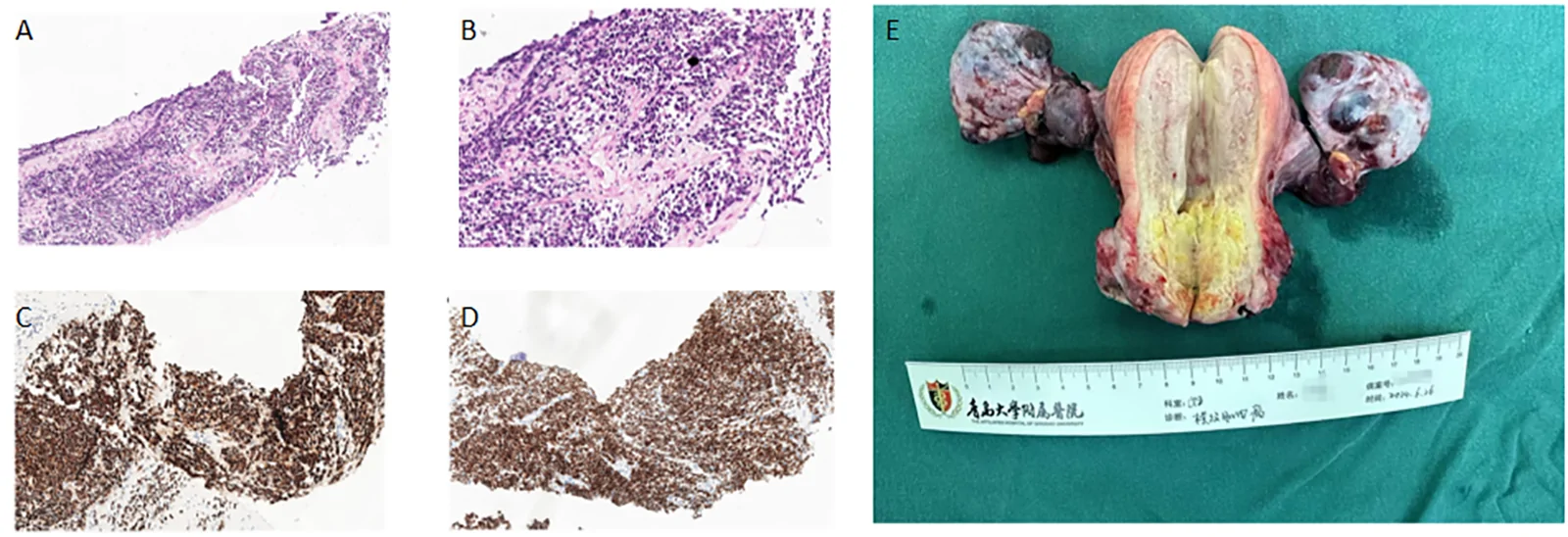
Gross specimen, pre-treatment biopsy histology and post-treatment surgical pathology of Patient B’s cervical embryonal rhabdomyosarcoma. **(A, B)** Hematoxylin-eosin (HE) staining of the initial pre-treatment transvaginal core needle biopsy (medium and high magnification), showing typical small round malignant tumor cells consistent with embryonal rhabdomyosarcoma; **(C)** Positive Myogenin immunohistochemical staining from pre-treatment biopsy tissue; **(D)** Positive MyoD1 immunohistochemical staining from pre-treatment biopsy tissue; **(E)** Gross appearance of the total hysterectomy specimen collected after neoadjuvant chemotherapy (pathological complete response, no residual viable tumor identified in surgical tissue).

On June 27, 2024, the patient underwent open radical hysterectomy, bilateral salpingo-oophorectomy, pelvic and para-aortic lymphadenectomy, and omentectomy. Postoperative pathology demonstrated no definitive residual malignant cells in the hysterectomy specimen, consistent with treatment-related regression following neoadjuvant chemotherapy (**Figure 4**). Immunohistochemistry on residual tissue was negative for myogenic markers: MyoD1 (−), Myogenin (−), Desmin (−). No malignancy was identified in any dissected lymph nodes. Notably, the pre-treatment transvaginal core needle biopsy had demonstrated positive myogenic staining (MyoD1+, Myogenin+), confirming the initial diagnosis of embryonal rhabdomyosarcoma. Postoperative diagnosis: Cervical embryonal rhabdomyosarcoma, stage IVB (T3N1M1), with metastases to pelvic, para-aortic, right cardiophrenic, and left supraclavicular lymph nodes; hydrosalpinx; history of tubal anastomosis and cesarean delivery. The patient achieved pathological complete response (pCR) following neoadjuvant VAC chemotherapy.

Postoperatively, two cycles of adjuvant VAC chemotherapy plus TOMO radiotherapy and vaginal brachytherapy were administered while awaiting molecular testing results. Comprehensive panel sequencing of postoperative tumor tissue identified five somatic aberrations with no pathogenic germline alterations. The tumor exhibited low tumor mutation burden (TMB-L, 0 Mut/Mb) and microsatellite stability (MSS), predicting limited benefit from immune checkpoint inhibitors. Two somatic missense mutations were detected: ARID2 c.1454C>T (p.A485V) and LRP2 c.10363C>T, both classified as Grade III variants without proven predictive value. Three somatic copy number variations were identified: FANCA whole-exon deletion (copy number 1.2, Grade II), MDM4 gene amplification (copy number 3.0, Grade II), and CDH1 whole-exon deletion (copy number 1.2, Grade III). Among these, the FANCA deletion was the sole homologous recombination repair (HRR) pathway alteration with theoretical therapeutic relevance, though formal HRD testing was not performed to confirm functional deficiency.

Given the absence of high-evidence actionable targets, oral olaparib 300 mg twice daily was initiated as exploratory maintenance therapy in February 2025 following adjuvant chemoradiotherapy. Olaparib monotherapy continued until June 2025, when the patient developed fever and severe thrombocytopenia (11 × 10^9^/L). Bone marrow aspiration confirmed tumor infiltration, and PET-CT demonstrated extensive skeletal hypermetabolism (skull, vertebrae, pelvis, bilateral extremities) with new hypodense hepatic (SUVmax 9.1) and pancreatic (SUVmax 8.6) metastases. No recurrence was detected in the original pelvic or retroperitoneal lymphatic regions. Following platelet transfusions and anti-infective supportive care, the patient requested transfer to a local facility for subsequent management, with follow-up maintained until August 2025.

## 3 Discussion

As briefly outlined in the Introduction, RMS ranks the most prevalent soft tissue sarcoma among children and adolescents, with ERMS as its dominant histological subtype (60% of all RMS cases) (7). WHO classification divides RMS into four histologic categories, and adult cervical ERMS remains extremely rare with poorer long-term survival than pediatric counterparts (1, 2, 8).The head, neck, and urogenital tract are the most common sites of embryonal RMS (9). Embryonal rhabdomyosarcomas (ERMS) have an incidence rate of less than 0.01% in children and adolescents, accounting for more than 50% of pediatric soft tissue sarcomas, whereas they are extremely rare in adults, accounting for only 1%–5% of adult soft tissue sarcomas (2, 7).The overall 5-year survival rate for children is approximately 70%–85%, whereas for adults or elderly patients, the 5-year survival rate is often <50%. The oldest reported case of embryonal rhabdomyosarcoma was a 76-year-old patient with uterine body embryonal rhabdomyosarcoma (10).Cervical embryonal rhabdomyosarcoma is relatively rare and typically occurs at a later age, primarily affecting young women aged 12 to 26 (9, 11). Approximately one-third of cases involve women aged 20 and older (12).

The characteristic histopathological features of embryonal rhabdomyosarcoma of the cervix include a germinal layer formed by the accumulation of primitive blue spindle-shaped or oval cells beneath the epithelium, areas of rhabdomyoblast differentiation, areas of anaplasia, and areas of chondroblastic differentiation. Both patients exhibited consistent immunohistochemical profiles typical of ERMS: diffuse Myogenin and focal MyoD1 positivity confirmed myogenic differentiation (3, 13, 14), while epithelial, lymphoid, neuroendocrine and melanocytic markers were entirely negative, verifying the mesenchymal origin of the tumor. High Ki-67 proliferation indices (80%–90%) in both lesions further indicated its highly invasive biological phenotype (3, 14, 15).According to reports, among histopathological features, foci of chondroid differentiation are a favorable prognostic factor, whereas deep muscle layer invasion, lymphatic invasion, and focal alveolar patterns are associated with poor prognosis (9).No cartilage or chondrocyte-like components were observed in the pathology of either patient, and neither patient had a DICER1 mutation; these are classified as sporadic, highly aggressive cases, which overall suggest a poor prognosis.

The clinical features of embryonal cervical rhabdomyosarcoma lack typicality. In this series, both cases of adult embryonal cervical rhabdomyosarcoma presented with abnormal vaginal bleeding as the primary symptom, manifesting as persistent vaginal bleeding and menorrhagia, respectively (3, 4). In the literature, other reported cases have also exhibited symptoms such as abdominal pain, dyspareunia, and postcoital bleeding (7, 9).Gynecological examination reveals a grape-like mass on the cervix (16) or a firm cervix with an enlarged, fixed uterus. The tumors are all massive, highly vascularized lesions, and β-HCG levels are negative, ruling out gestational trophoblastic disease. In the advanced stage of the disease, patients may experience lymph node metastasis and distant metastasis. RMS predominantly spreads hematogenously (17, 18), and distant metastasis correlates with tumor size, site and histology, predicting poorer survival (19). Combined immunohistochemical staining, molecular detection and cross-sectional imaging facilitate accurate tumor staging (20–23).

Currently, no disease-specific international guidelines exist for adult cervical ERMS, and current management is largely extrapolated from pediatric rhabdomyosarcoma protocols and NCCN soft tissue sarcoma recommendations (1, 5); integrated surgical, chemotherapeutic and radiotherapeutic interventions are widely adopted for clinical management owing to the tumor’s inherent chemosensitivity (11, 24–29).

The primary goals of surgical treatment are complete resection of the primary tumor and achievement of negative surgical margins (30). The extent of residual disease post-surgery is the most important factor influencing prognosis (31, 32). The traditional standard procedure consists of radical hysterectomy + bilateral salpingo-oophorectomy + pelvic and para-aortic lymph node dissection (1).The first-line chemotherapy regimen is VAC (a combination of vincristine, actinomycin D, and cyclophosphamide) (9), which is the internationally recognized standard chemotherapy regimen (31). The VIA regimen (combining ifosfamide, vincristine, and actinomycin D) is also widely used in Europe; neoadjuvant chemotherapy can reduce the size of advanced lesions and improve the rate of surgical resection (30), while postoperative adjuvant chemotherapy can eliminate micrometastases and reduce the risk of recurrence (30). For patients with recurrence or distant metastasis, systemic combination chemotherapy is the primary control strategy (11, 26–29). Radiotherapy, as a local adjuvant treatment (5), is indicated for patients with positive surgical margins or residual disease; when used in younger patients, the potential for reproductive function impairment must be carefully weighed. Furthermore, disease relapse risk in rhabdomyosarcoma correlates tightly with chemotherapy adherence, histological subtype and depth of primary tumor invasion (30, 31). For patients with solitary recurrent lesions, surgical resection remains a viable intervention, and prolonged routine surveillance is mandatory post-treatment to detect recurrent disease and monitor chronic treatment-related complications (1).

Notably, PARP inhibitors are not standard therapy for rhabdomyosarcoma and represent an off-label, individualized approach reserved for patients with targetable genomic alterations. Olaparib maintenance was administered in Patient B solely based on the detection of a somatic FANCA deletion (33, 34). Although PARP inhibitors have theoretical antitumor activity in advanced malignancies, single-agent olaparib affords limited long-term disease control, particularly in the absence of standardized HRD testing to guide patient selection. Olaparib was given at 300 mg twice daily—the FDA-approved monotherapy dose for HRR-deficient epithelial tumors—as no formal dosing recommendations exist for soft tissue sarcomas harboring FANCA loss.

Both patients ultimately developed systemic progression, attributable to multiple interrelated factors. First, adult cervical ERMS exhibits highly aggressive biology, with exceedingly high Ki-67 indices (80%–90%) and no favorable chondroid differentiation; both cases were diagnosed at stage IVB with extensive synchronous lymphatic, pulmonary, osseous, and visceral metastases. Large-volume systemic disease and occult bone marrow micrometastases cannot be eradicated by local surgery and radiotherapy alone. Second, primary and acquired chemoresistance emerged: Patient A failed multiple salvage regimens after VAC progression, while Patient B, despite achieving pelvic pCR with neoadjuvant VAC, harbored chemoresistant subclones beyond the pelvic cavity. Third, olaparib maintenance relied solely on an isolated FANCA deletion with low-level predictive evidence; standardized HRD testing was not performed, and no additional actionable targets were identified, resulting in insufficient sustained tumor suppression. Fourth, cumulative myelosuppression from chemotherapy and targeted therapy led to poor tolerance and incomplete treatment cycles. Collectively, the inherent tumor aggressiveness, extensive metastatic burden at diagnosis, chemoresistance, limitations of off-label targeted therapy, and treatment-related toxicity jointly contributed to the failure of long-term disease control.

## 4 Conclusion

We report two reproductive-age women with stage IVB cervical ERMS characterized by abnormal vaginal bleeding, enlarged uterus, cervical mass and normal serum β-hCG; both patients eventually developed systemic tumor progression despite standardized treatment. Compared with Patient A, Patient B derived a longer survival benefit from neoadjuvant chemotherapy followed by radical surgery and sequential adjuvant chemoradiotherapy. Given the highly aggressive biological behavior of this rare neoplasm, patients with advanced cervical ERMS should receive personalized combined therapy formulated via cross-disciplinary collaboration to optimize long-term survival prognosis.

## 5 Patient perspective

The patient’s family members gave their informed consent to the treatment plan and expressed deep gratitude to the team for their precise diagnosis and treatment as well as their comprehensive management throughout the process. The first-line chemotherapy effectively controlled the condition. Although the targeted therapy showed progression later on, the entire treatment process was implemented with reference to existing consensus for rare gynecological sarcomas, maximizing the clinical benefits for the patient.

## Data Availability

The original contributions presented in the study are included in the article/supplementary material. Further inquiries can be directed to the corresponding author.
